# High-Performance Aqueous Zinc-Ion Batteries Realized by MOF Materials

**DOI:** 10.1007/s40820-020-00487-1

**Published:** 2020-07-17

**Authors:** Xuechao Pu, Baozheng Jiang, Xianli Wang, Wenbao Liu, Liubing Dong, Feiyu Kang, Chengjun Xu

**Affiliations:** 1 Shenzhen Geim Graphene Center, Tsinghua Shenzhen International Graduate School, Tsinghua University, Shenzhen, 518055 People’s Republic of China; 2 State Key Laboratory of New Ceramics and Fine Processing, School of Materials Science and Engineering, Tsinghua University, Beijing, 100084 People’s Republic of China; 3 Tsinghua-Berkeley Shenzhen Institute (TBSI), Tsinghua University, Shenzhen, 518055 People’s Republic of China; 4 College of Chemistry and Materials Science, Jinan University, Guangzhou, 511443 People’s Republic of China

**Keywords:** Zinc-ion battery, Metal–organic framework, Cathode material, Zn anode

## Abstract

Rechargeable aqueous zinc-ion batteries (ZIBs) have been gaining increasing interest for large-scale energy storage applications due to their high safety, good rate capability, and low cost. However, the further development of ZIBs is impeded by two main challenges: Currently reported cathode materials usually suffer from rapid capacity fading or high toxicity, and meanwhile, unstable zinc stripping/plating on Zn anode seriously shortens the cycling life of ZIBs. In this paper, metal–organic framework (MOF) materials are proposed to simultaneously address these issues and realize high-performance ZIBs with Mn(BTC) MOF cathodes and ZIF-8-coated Zn (ZIF-8@Zn) anodes. Various MOF materials were synthesized, and Mn(BTC) MOF was found to exhibit the best Zn^2+^-storage ability with a capacity of 112 mAh g^−1^. Zn^2+^ storage mechanism of the Mn(BTC) was carefully studied. Besides, ZIF-8@Zn anodes were prepared by coating ZIF-8 MOF material on Zn foils. Unique porous structure of the ZIF-8 coating guided uniform Zn stripping/plating on the surface of Zn anodes. As a result, the ZIF-8@Zn anodes exhibited stable Zn stripping/plating behaviors, with 8 times longer cycle life than bare Zn foils. Based on the above, high-performance aqueous ZIBs were constructed using the Mn(BTC) cathodes and the ZIF-8@Zn anodes, which displayed an excellent long-cycling stability without obvious capacity fading after 900 charge/discharge cycles. This work provides a new opportunity for high-performance energy storage system.

**Electronic supplementary material:**

The online version of this article (10.1007/s40820-020-00487-1) contains supplementary material, which is available to authorized users.

## Highlights

- Various MOF materials were synthesized and investigated as ZIB cathodes.
- A long-term stable ZIF-8@Zn anode was proposed by coating ZIF-8 material on the surface of zinc foils.
- High-performance aqueous ZIBs were constructed using the Mn(BTC) cathode and the ZIF-8@Zn anode.

**Electronic supplementary material:**

The online version of this article (10.1007/s40820-020-00487-1) contains supplementary material, which is available to authorized users.

## Graphical abstract

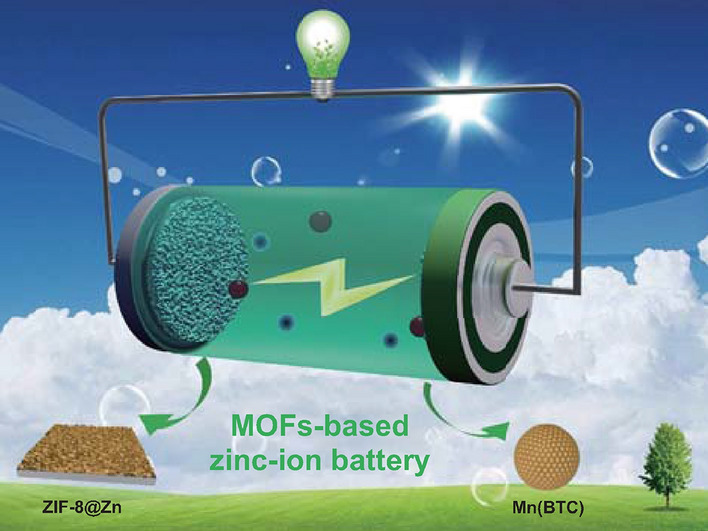

## Introduction

Lithium-ion batteries have been widely used in modern society [1]. However, security problems and increasing production cost pose challenges for their large-scale applications in the field of electric vehicles [2–4]. Researchers are committed to seeking alternative electrochemical energy storage systems to lithium-ion batteries. Many new-type rechargeable batteries using multivalent metal ions (e.g., Zn^2+^, Mg^2+^, and Al^3+^) as charge carriers have been proposed successively [5–7]. Among them, rechargeable aqueous zinc-ion batteries (ZIBs) have been considered as highly promising candidate for the next-generation energy storage system, because they are safe, low cost, and environmental benign [5]. In order to meet the demands of common energy consumption, two main issues should be addressed for the ZIBs. One is exploring suitable cathode materials for the reversible intercalation/extraction of zinc-ions [8, 9], and the other is exploring stable zinc metal anodes because this is essential for ZIBs to realize a long cycle life [10–13].

To find high-performance cathode materials for ZIBs, many attempts have been made [14]. Up to now, several types of ZIB cathode materials have been reported, including Mn-based materials [15–22], V-based materials [23–29], Prussian blue analogs [30–32], and some other cathode materials such as Mo_6_S_8_ [33], quinone [34], and poly(benzoquinonyl sulfide) [35]. Mn-based materials, especially manganese oxides, possess the advantages such as low cost and environmental friendliness, but they suffer from the problems of rapid capacity fading and poor rate performance [36]. V-based cathode materials generally exhibit superior Zn^2+^-storage ability [37, 38], whereas serious toxicity limits their large-scale applications for ZIBs [10]. The Zn^2+^-storage capacity of Prussian blue analogs is only about 50 mAh g^−1^, which determines that the Prussian blue analogs may be not qualified to construct high-energy ZIBs [30]. In view of the above discussion, developing high-performance cathode materials for ZIBs is still a challenge. Furthermore, metallic Zn electrode owns an ultrahigh volumetric capacity and low redox potential (− 0.76 V vs. standard hydrogen electrode), which enable it to be a promising anode for ZIBs [5, 39, 40]. However, in practical applications, repeated deposition/dissolution of Zn inclines to form zinc dendrites/protuberances, leading to severe polarization and a short circuit of the batteries [41]. Some modification strategies such as introducing additives, employing gel electrolyte, using 3D current collectors, and creating the protection layer have been proposed to regulate Zn stripping/plating behaviors and realized dendrite-free zinc metal anodes [11, 12, 42, 43]. In a word, stabilizing zinc anodes is also critical to realize long-life ZIBs.

Exploring new potential materials such as metal-organic frameworks (MOFs) may open opportunities for addressing these problems. MOFs are characterized by highly porous structure, designable frameworks, and multifunctionality [44–46]. In the past two decades, MOFs have been widely used in energy storage systems such as lithium-ion batteries [47], supercapacitors [48], and fuel cells [49]. Recently, MOFs are attracting increasing interests in the field of ZIBs. For instance, a MnO_*x*_/N-doped carbon cathode material was synthesized based on a MOF template and provided a Zn^2+^-storage capacity of 305 mAh g^−1^ even after 600 charge/discharge cycles [36]. Besides, a MOF-based single-ion Zn^2+^ solid-state electrolyte with high ionic conductivity, high Zn^2+^ transference number, and good electrochemical stability was designed to achieve dendrite-free Zn batteries [50]. These researches imply that MOFs may provide new opportunities to construct high-performance ZIBs.

Herein, we synthesized five kinds of MOFs materials, including Mn(BTC), Mn(BDC), Fe(BDC), Co(BDC), and V(BDC) (in which BDC is 1,4-dicarboxybenzene and BTC is 1,3,5-benzenetricarboxylic acid.) and investigated their electrochemical behaviors as ZIB cathodes. Among these MOFs materials, Mn(BTC) showed the best Zn^2+^ storage capability. Zn^2+^ storage mechanism of the Mn(BTC) cathode was then comprehensively studied. Besides, we developed a long-term stable ZIF-8@Zn anode by coating ZIF-8 material on the surface of zinc foils. Furthermore, high-performance aqueous ZIBs were constructed using the Mn(BTC) cathodes and the ZIF-8@Zn anodes, which exhibited an excellent cycling stability with 92% capacity retention after 900 charge/discharge cycles. This work opens up a new door for achieving high-performance aqueous zinc-ion batteries based on MOFs materials.

## Experimental Section

### Synthesis of MOF Materials

To synthesize Mn(BTC), 1225 mg Mn(CH_3_COO)_2_·4H_2_O and 300 mg polyvinyl pyrrolidone (PVP) were dissolved in ethanol/H_2_O (125/125 mL) to get solution A, and meanwhile, 2250 mg trimesic acid (H_3_BTC) was dissolved in another ethanol/H_2_O (125/125 mL) system to get solution B. Then, solution B was added slowly into solution A under continuous stirring. After 10 min, the reaction mixture was aged without interruption for 24 h. The products were collected after centrifugation, washing several times with ethanol and complete drying in an oven at 60 °C. Fe(BDC), Mn(BDC), Co(BDC), and V(BDC) were synthesized according to previously reported methods [51–55].

### Preparation of ZIF-8@Zn Anode

ZIF-8 was purchased from J&K Scientific Ltd. The as-received ZIF-8 and polyvinylidene fluoride (PVDF) were mixed with a mass ratio of 8:2 in *N*-methyl-2-pyrrolidone (NMP) solvent to form a homogeneous slurry. Then, the slurry was uniformly coated onto a Zn foil and dried at 80 °C overnight in a vacuum to obtain the ZIF-8-coated Zn (i.e., ZIF-8@Zn) anodes, in which the mass of the ZIF-8 coating was about 1.1 mg cm^−2^.

### Material Characterization

Crystallographic and structure analysis was carried out by X-ray diffraction (XRD, Rigaku 2500, Cu Kα radiation, *λ* = 0.154056 nm) with a scan rate of 5° min^−1^ over 2-theta ranging from 5° to 70°. Field emission scanning electron microscopy (FE-SEM) was performed on a Zeiss Supra55 scanning electron microscope. Elemental analysis was characterized by energy-dispersive X-ray spectroscopy (EDS) on the FE-SEM. X-ray photoelectron spectroscopy (XPS, ESCALAB 250X, Thermo Fisher, United Kingdom) was used for characterizing the valence variation. Fourier transform infrared (FTIR, Thermo Scientific Nicolet iS 50) spectroscopy was used to identify functional groups in the MOF materials at pristine state and various charge/discharge states. Element content in electrolytes was analyzed by inductively coupled plasma atomic emission spectrometry (ICP-AES).

### Electrochemical Measurements of MOF Cathodes

The electrochemical performance of various MOF cathodes was evaluated in CR2032 coin cells with zinc foil anode, air-laid paper separator, and 2 M ZnSO_4_ aqueous electrolyte. To prepare the MOF cathodes, the synthesized MOF powder was mixed with acetylene black and PVDF with a weight ratio of 7:2:1 in NMP solvent and then coated onto a stainless-steel current collector and dried at 80 °C overnight. The mass loading of MOF materials on current collectors is about 1.0 mg cm^−2^. For the assembled cells, cyclic voltammetry (CV) tests were carried out on a VMP3 multichannel electrochemical station (Bio-Logic Science Instruments SA) at a sweep rate of 0.5 mV s^−1^, and galvanostatic charge–discharge (GCD) tests were performed on a LAND CT2001 battery tester at current density of 50 mA g^−1^.

### Electrochemical Measurements of ZIF-8@Zn Anodes

Electrochemical behaviors of the ZIF-8@Zn anodes were characterized through ZIF-8@Zn||ZIF-8@Zn symmetrical cells, in which both the negative and positive electrodes were a ZIF-8@Zn disk (with diameter of 12 mm), air-laid paper was used as the separator, and 2 M ZnSO_4_ (or 2 M ZnSO_4_ + 0.1 M MnSO_4_) aqueous solution was used as the electrolyte. For comparison, Zn||ZnSO_4_||Zn symmetrical cells were also assembled, in which pure Zn foils served as Zn electrodes. Electrochemical stability of these symmetric cells was evaluated by GCD tests at different current densities of 0.25–2 mA cm^−2^ and different charge/discharge capacities of 0.05–0.4 mAh cm^−2^ on a LAND CT2001 battery tester. The impedance measurements of symmetrical cells were taken on a VMP3 multichannel electrochemical station (Bio-Logic Science Instruments SA) between 300 kHz and 0.01 Hz.

### Construction and Electrochemical tests of Mn(BTC) Cathode//ZIF-8@Zn Anode ZIBs

The MOF//ZIF-8@Zn ZIBs were constructed based on the Mn(BTC) cathode, ZIF-8@Zn anode, an air-laid paper separator, and 2 M ZnSO_4_ (or 2 M ZnSO_4_ + 0.1 M MnSO_4_) electrolyte. CV and GCD measurements were taken on the Bio-Logic VMP3 electrochemical station and the LAND CT2001 battery tester, respectively.

## Results and Discussion

### Material Characterization and Electrochemical Properties of MOF Cathodes

**Fig. 1:**
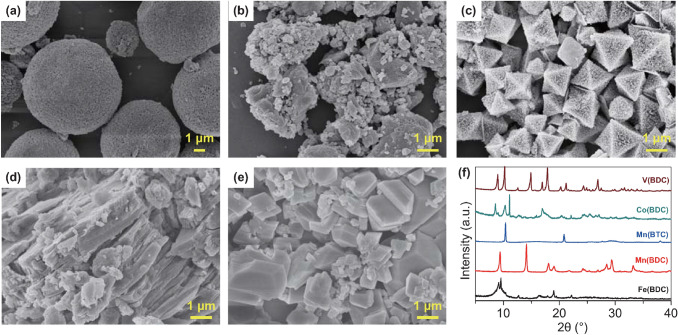
SEM images of the synthesized MOF materials: **a** Mn(BTC), **b** Mn(BDC), **c** Fe(BDC), **d** Co(BDC), and **e** V(BDC), **f** XRD patterns of the samples

Structure and morphology of the as-synthesized MOF materials including Mn(BTC), Mn(BDC), Fe(BDC), Co(BDC), and V(BDC) were characterized by X-ray diffraction (XRD) and scanning electron microscope (SEM). Figures 1a–e and S1 display that the Mn(BTC) and Fe(BDC) are composed of spherical and octahedral particles with size of 1–10 μm, respectively, and the other three MOF samples have irregular-shaped micromorphologies. As shown in Fig. 1f and Fig. S2, the XRD patterns of the synthesized MOF materials are consistent with previously reported literature [51–55], indicating that Mn(BTC), Mn(BDC), Fe(BDC), Co(BDC), and V(BDC) MOFs were successfully synthesized. The crystalline structures of these MOF materials are depicted in Fig. S3.

**Fig. 2:**
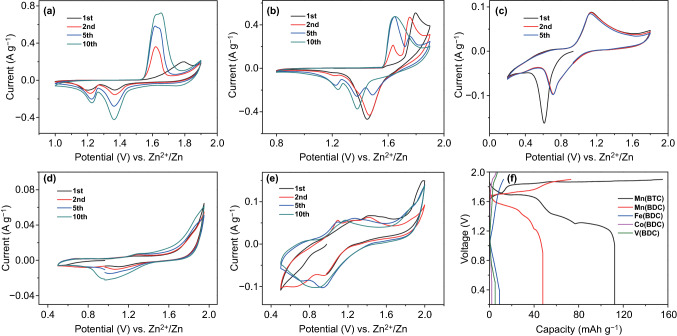
CV curves at 0.5 mV s^−1^ of the MOF cathodes: **a** Mn(BTC), **b** Mn(BDC), **c** Fe(BDC), **d** Co(BDC), and **e** V(BDC), **f** Charge/discharge curves at 50 mA g^−1^ of the MOF cathodes

To evaluate Zn^2+^-storage ability of the above MOF materials as ZIB cathodes, cyclic voltammetry (CV) and galvanostatic charge–discharge (GCD) tests were conducted in 2 M ZnSO_4_ aqueous electrolyte. These MOF materials are selected as ZIB cathodes because the transition metal centers of Mn, Fe, Co, and V have proved to be active sites in electrochemical energy storage systems [56–60]. Meanwhile, BDC and BTC ligands are conventional ligands for MOF materials, and their lightweight feature is beneficial for the MOF materials to achieve a higher theoretical capacity considering that ligands usually cannot be redox sites for metal-ion storage [61]. Corresponding CV curves are shown in Fig. 2a–e. For the Mn(BTC) cathode, it can operate in a voltage window of 1.0–1.9 V (vs. Zn^2+^/Zn), and reversible redox peaks are observed on the CV curves (Fig. 2a), preliminarily demonstrating effective Zn^2+^ storage in the Mn(BTC). For the CV curves of the Mn(BDC) and Fe(BDC) MOF cathodes in Fig. 2b, c, there also emerge 1–3 pairs of reversible redox peaks, but the peak currents of the Fe(BDC) MOF are much smaller. The redox peaks in the CV curves of the Co(BDC) and V(BDC) are not obvious, and response currents are very small (Fig. 2d, e), suggesting a poor Zn^2+^-storage ability. Figure 2f displays charge/discharge curves at a current of 50 mA g^−1^ of the five MOF cathode materials. The Mn(BTC) exhibits several voltage plateaus in its discharge curve and delivers the highest Zn^2+^-storage capacity of 112 mAh g^−1^. Specific capacity of the Mn(BDC) cathode is about 48 mAh g^−1^. In contrast, the other three MOF cathodes including Fe(BDC), Co(BDC), and V(BDC) are incapable of effectively storing Zn^2+^, which is reflected by their very low capacities of less than 10 mAh g^−1^. Obviously, for the above MOF samples, only Mn(BTC) is promising to be utilized as a high-performance cathode material for ZIBs. Despite this, the Mn(BTC) shows a modest cycling stability during repeated Zn^2+^ storage-release processes (Fig. S4). This issue needs to be resolved based on the reveal of Zn^2+^-storage mechanism of the Mn(BTC) cathode.

### Energy Storage Mechanism of the Mn(BTC) Cathode

**Fig. 3:**
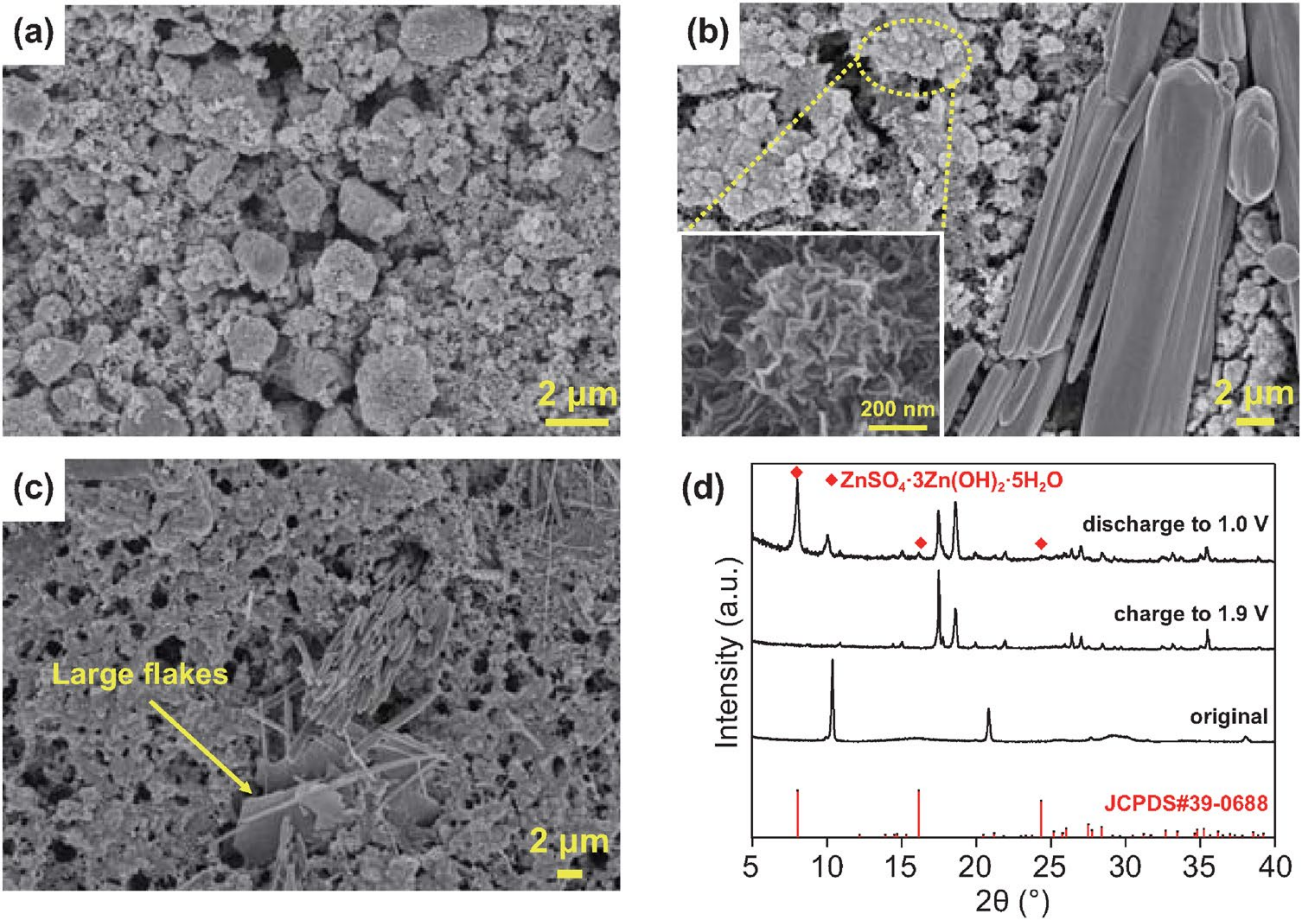
SEM images of Mn(BTC) cathode at different states: **a** original state, **b** charge to 1.9 V and **c** discharge to 1.0 V. Inset in **b** is a high-resolution SEM image of the encircled zone. **d** XRD patterns of the cathode at the above states

Zn^2+^-storage mechanism of the Mn(BTC) cathode in ZnSO_4_ electrolyte is further investigated. Evolution of the phase composition and micromorphology of the Mn(BTC) electrode during Zn^2+^ storage-release processes were characterized by XRD and SEM, as shown in Fig. 3. When the fresh Mn(BTC) cathode is charged to 1.9 V at 50 mA g^−1^, “nanoflower-like” and “rod-like” particles appear (Fig. 3b). The “nanoflower” particles densely distribute on the surface of the electrode. Energy-dispersive X-ray spectroscopy (EDS) analysis in Fig. S5 points out that the “nanoflower” particles contain Mn and O elements, and the main composition elements of the “rod-like” particles are C, O, and Zn. When the Mn(BTC) cathode is then discharged to 1.0 V (Fig. 3c), the “rod-like” particles still exist, but the “nanoflower-like” particles disappear, and meanwhile, some large flakes form. The XRD results of the cathode at different charge–discharge states are shown in Fig. 3d. Compared with the original state, XRD pattern of the Mn(BTC) cathode at the fully charged state (i.e., 1.9 V) shows obvious differences. The main peaks (at 10.4°, 20.8°, 29.4°, 38.0°, and 42.4°) of the Mn(BTC) disappear, and some new peaks emerge (at 17.5°, 18.6°, 21.9°, 26.4°, 27.0°, and 35.5°), which means that there arises a phase transition reaction and new phases form undergoing charging. XRD pattern at the fully discharged state (i.e., 1.0 V) shows the characteristic diffraction peaks of ZnSO_4_·3Zn(OH)_2_·5H_2_O (denoted as BZSP). Therefore, the above-mentioned large flakes in Fig. 3c are considered as BZSP. Besides, from the fully charged state to the fully discharged state, some diffraction peaks (e.g., at 17.5°, 18.6°, 26.4°, 27.0°, and 35.5°) of the cathode remain unchanged positions. No PDF cards of MnO_2_ can match well with these diffraction peaks, and they still remain their positions at the fully discharged state, so these diffraction peaks do not belong to MnO_2_. These diffraction peaks are thought to originate from the “rod-like” particles that are observed in Fig. 3b, c.

**Fig. 4:**
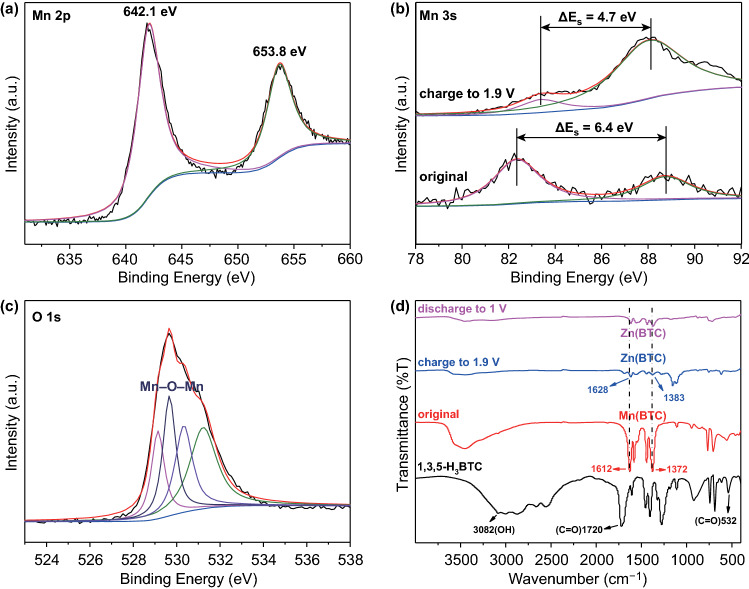
XPS spectra of the Mn(BTC) cathode at fully charged state: **a** Mn 2*p*, **b** Mn 3*s*, and **c** O 1*s*. Mn 3*s* XPS spectrum of the original Mn(BTC) cathode is also presented in **b**. **d** FTIR spectra of 1,3,5-H_3_BTC, original Mn(BTC) cathode, Mn(BTC) cathode at 1.9 and 1.0 V

To figure out what the “rod-like” and “nanoflower-like” compounds (Fig. 3b, c) are, X-ray photoelectron spectroscopy (XPS) and Fourier transform infrared spectroscopy (FTIR) were further carried out in Fig. 4. The high-resolution Mn 2*p* XPS spectrum of the Mn(BTC) cathode at fully charged state (Fig. 4a) shows that the Mn 2*p*_3/2_ and Mn 2*p*_1/2_ peaks situate at 642.1 and 653.8 eV, respectively, with the peak separation of 11.7 eV. These values are consistent with the reported parameters for MnO_2_ [62–65]. Considering that these “nanoflower-like” particles contain Mn element while “rod-like” particles do not (as discussed in Fig. S5a), the “nanoflower-like” particles are determined to be MnO_2_. In Fig. 4b, peak separation of Mn 3*s* orbit decreases from 6.4 eV for the original Mn(BTC) cathode to 4.7 eV for the fully charged Mn(BTC) cathode, indicating the increase in the Mn oxidation state after charging process [19]. The O 1*s* core-level spectrum in Fig. 4c can be divided into four main peaks. Especially, the peak centered at 529.7 eV is in accord with the typical bond of Mn–O–Mn [66].

FTIR spectra of the Mn(BTC) cathode at original, charging, and discharging states are presented in Fig. 4d. In the FTIR spectrum of the original Mn(BTC), the absorption peaks of O–H at 3082 cm^−1^ and C=O at 1720 cm^−1^ from 1,3,5-benzenetricarboxylic acid disappear, while the asymmetric stretching vibrations of –COO– and symmetric stretching vibrations of –COO– are detected in the regions of 1612–1545 and 1433–1372 cm^−1^, respectively. This is an evidence that the manganese ions have been successfully coordinated with the 1,3,5-BTC ligands in the Mn(BTC) material [56]. When the Mn(BTC) is charged to 1.9 V, the asymmetric stretching vibration of –COO– at 1612 cm^−1^ shifts to a new band at 1628 cm^−1^. Meanwhile, the symmetric stretching vibration of –COO– at 1372 cm^−1^ shifts to a new band at 1383 cm^−1^. They are coordination characteristic bands of Zn(BTC) MOF materials [67]. These suggest that zinc-ion substitute manganese-ion to be coordinated with -COOH to form Zn(BTC). When the Mn(BTC) cathode is then discharged to 1.0 V, there is no distinct change for these typical bands, suggesting that Zn(BTC) remains stable during discharge process.

Based on the above analysis, we speculate possible reaction paths of the Mn(BTC) cathode during charge–discharge processes. During the first charge process, there occurs a transformation from Mn(BTC) to Zn(BTC), and Mn^2+^ dissolves into electrolyte. These Mn^2+^ is oxidized to MnO_2_ on the cathode surface through a normal manganese deposition reaction as the charging process proceeds. The reaction paths were further confirmed by plasma atomic emission spectrometry (ICP-AES) tests. As shown in Table S1, Mn element concentration in electrolyte was detected when the cathode was charged/discharged to various states. Mn element concentration in electrolyte is 6.15 and 4.62 mg L^−1^ when the cathode is charged to 1.8 and 1.9 V, respectively. Decrease in Mn concentration in electrolyte is attributed to manganese deposition reaction. Subsequently, the deposited MnO_2_ serves as a host for Zn^2+^ and H^+^ storage in the following charge/discharge processes, which is the reason that we detected the formation of BZSP during charge/discharge processes (Fig. 3c, d) [68]. It worth noting that the BTC organic ligands of Mn(BTC) cathodes are not involved in the Zn-storage redox process, not only because the BTC ligands have been proved to be redox-innocent ligands [61] but also because they are always detected during charge/discharge processes of our ZIB systems (which means that BTC would not directly participate in Zn-storage reactions). However, BTC ligand is an indispensable part of Mn(BTC) because it constructs the three-dimensional framework of MOF materials. Interestingly, as shown in Fig. S6, Zn(BTC) particles seem to be the main by-product in this system and the formation of BZSP by-product is suppressed in this system compared with previously reported MnO_2_ cathode [68]. Unlike densely arranged BZSP flakes generated in MnO_2_//Zn ZIBs [68], these rod-like Zn(BTC) particles (generated in Mn(BTC)//Zn ZIBs) do not impede the direct contact between electrolyte and electrode materials, which is beneficial to ions diffusion and prolonged cycle life of the ZIBs.

In the above-proposed reaction pathways, the oxidation reaction from Mn(BTC) to MnO_2_ plays an important role in this system. On the one hand, MnO_2_ is capable of delivering a high capacity, but on the other hand, MnO_2_ generally suffers from rapid capacity fading due to the dissolution of manganese from the MnO_2_ electrode [15]. Therefore, the poor cycling stability of the Mn(BTC)//ZnSO_4_//Zn system as discussed in Fig. S4 is considered to be mainly caused by the dissolution of MnO_2_.

### MOF Material Stabilized Zn Metal Anodes

**Fig. 5:**
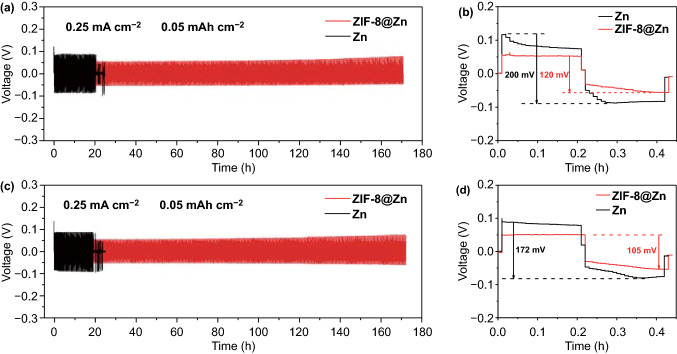
Cycling performance of symmetrical cells with bare Zn foil and ZIF-8@Zn electrodes in **a** and **b** 2 M ZnSO_4_ and **c** and **d** 2 M ZnSO_4_ + 0.1 M MnSO_4_ electrolytes. Applied current density is 0.25 mA cm^−2^ with Zn deposition/dissolution capacity of 0.05 mAh cm^−2^. **b**, **d** The first-cycle voltage profiles of symmetrical cells with bare Zn foil and ZIF-8@Zn electrodes

To achieve stable zinc anodes, we introduced a ZIF-8 coating on bare Zn foil. Electrochemical stability of the ZIF-8-coated Zn foil electrode (i.e., ZIF-8@Zn) was characterized in symmetrical ZIF-8@Zn||ZIF-8@Zn coin cells at a current density of 0.25 mA cm^−2^ and a charge/discharge capacity of 0.05 mAh cm^−2^. Figure 5a shows the cycling performance of bare Zn foil electrode and the ZIF-8@Zn electrode based symmetric cells with 2 M ZnSO_4_ electrolyte. It can be seen that a sudden short circuit appears after ~ 20 h stripping/plating for bare Zn foil. By contrast, the ZIF-8@Zn electrode works stably for at least 170 h and corresponding polarization voltage almost keeps constant, implying that the ZIF-8@Zn electrode has superior cycle stability. Furthermore, compared with the first cycle charge/discharge profile of the bare Zn foil in Fig. 5b, the ZIF-8@Zn electrode demonstrates a lower polarization voltage (120 vs. 200 mV for the bare Zn foil-based symmetric cells), which indicates a low energy barrier for metal nucleation on the surface of the ZIF-8@Zn electrode [69]. Very similarly, in the electrolyte of 2 M ZnSO_4_ + 0.1 M MnSO_4_ mixture solution (Fig. 5c, d), the ZIF-8@Zn electrode-based symmetric cells exhibit small polarization voltages and a long-term stable charge/discharge behavior over 170 h, whereas the bare Zn foil electrode-based symmetric cells perform poorly. The remarkably improved cycling stability of the ZIF-8@Zn anode are also observed at larger current densities of 0.5–2 mA cm^−2^ and higher Zn deposited depth of 0.1–0.4 mAh cm^−2^ (Fig. S7). All batteries exhibit stable cycling life over 150 h, further confirming the benefits of the ZIF-8@Zn anode on Zn stripping/plating behaviors. Besides, electrochemical impedance spectroscopy (EIS) test was also conducted to study the interfacial properties of bare Zn and ZIF-8@Zn anodes. As shown in Fig. S8, bare Zn electrodes delivered a large interfacial resistance and charge transfer resistance (estimated from the semicircle arc at high frequency range), which is due to passivation surface layers on Zn foils [70]. By contrast, the ZIF-8@Zn electrodes show a smaller interfacial resistance since the ZIF-8 coating provides a more stable electrode interface to guide a uniform Zn stripping/plating process.

**Fig. 6:**
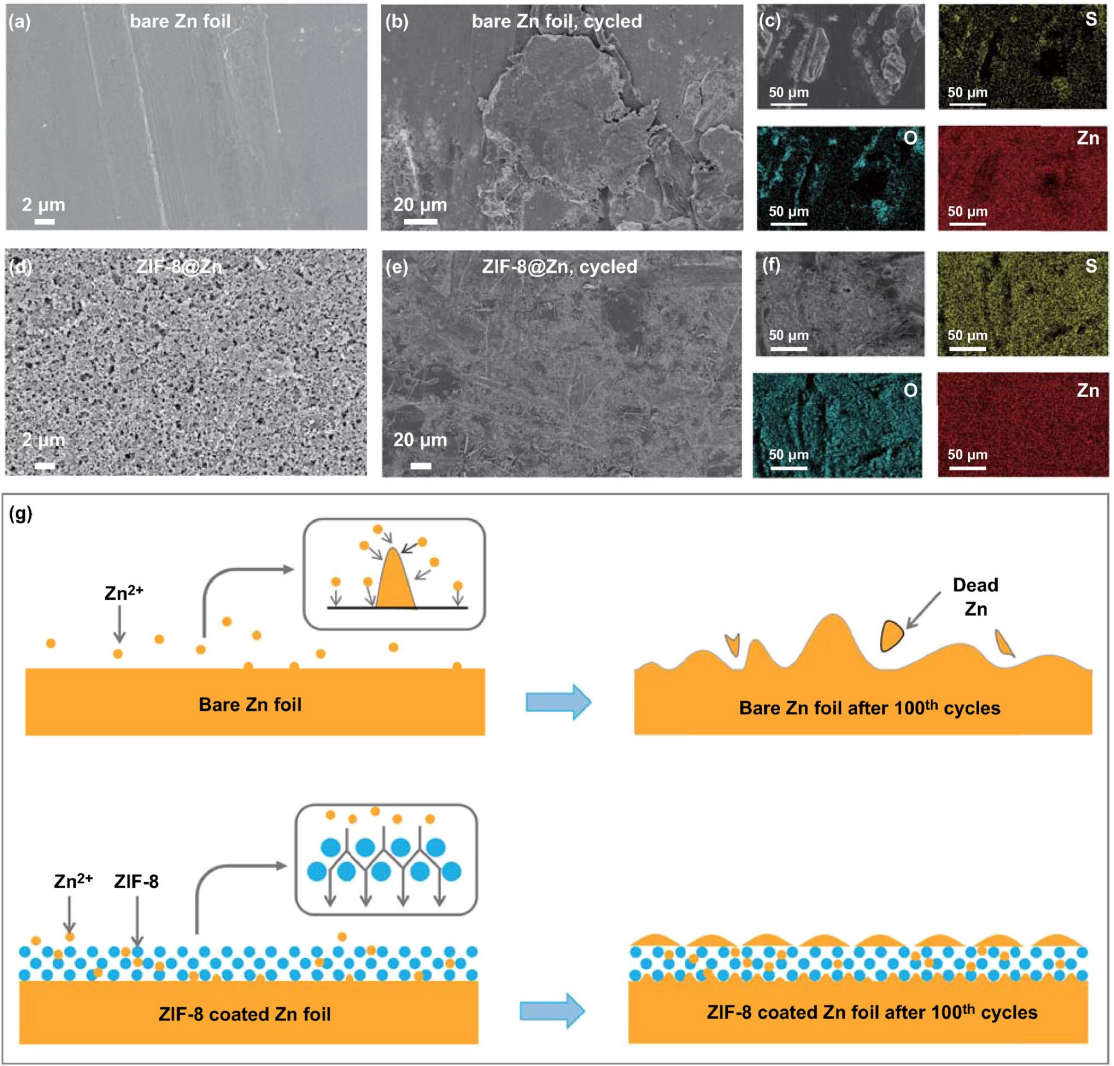
SEM images and EDS mapping of bare Zn electrode **a** before and **b**, **c** after 100 stripping/plating cycles, ZIF-8@Zn electrode **d** before, and **e**, **f** after 100 stripping/plating cycles. **g** Schematic illustration for morphology change of the bare Zn foil and ZIF-8@Zn electrodes during repeated Zn stripping/plating processes

Potential mechanism for the optimized electrochemical performance of the ZIF-8@Zn electrode is further investigated. We analyzed the morphology evolution of the bare Zn foil electrode and the ZIF-8@Zn electrode before and after 100 charge/discharge cycles by SEM and EDS mapping. As shown in Fig. 6a, b, many large protuberances appear on the surface of the Zn foil after cycling, suggesting uneven zinc plating/stripping process during repeated charge/discharge cycles. EDS mapping in Fig. 6c and Fig. S9 demonstrates that these protuberances contain O and Zn elements; thus, they are zinc oxides/hydroxides, as reported in the literature [11]. Cross-sectional SEM image (Fig. S10a, b) further shows some dead Zn particles on the surface of the bare Zn foil after cycling. In Fig. 6d, we can see that porous ZIF-8 coating is on the surface of the ZIF-8@Zn electrode, and after cycling (Fig. 6e, f), no large protuberances/dendrites appear. Besides, cross-sectional SEM and EDS images of the ZIF-8@Zn electrode (Fig. S10c, d) show that ZIF-8 layer does not fall off the Zn foil after 100 cycles, which means the ZIF-8 coating is stable in ZIBs. A schematic illustration for the morphology evolution of bare Zn and ZIF-8@Zn electrodes during repeated Zn stripping/plating is presented in Fig. 6g. According to the literature [71–74], Zn inclines to deposit on some sites to form small protuberances/dendrites during the plating process. These small protuberances/dendrites then generate inhomogeneous electric field, which is apt to attract Zn^2+^ to grow into uncontrolled protuberances/dendrites. When stripping, these large protuberances/dendrites incline to dissolve from their root positions, leading to the formation of “dead” Zn. Furthermore, the vigorously growing zinc protuberances/dendrites may easily pierce through the separator and cause a short circuit. For ZIF-8@Zn electrodes, the porous structure of ZIF-8 coating can homogenize the zinc-ion flux, avoiding an uneven distribution of the electric field and thus inhibiting the formation of protuberances/dendrites [75–77].

### ZIBs of Mn(BTC) Cathode//ZIF-8@Zn Anode

**Fig. 7:**
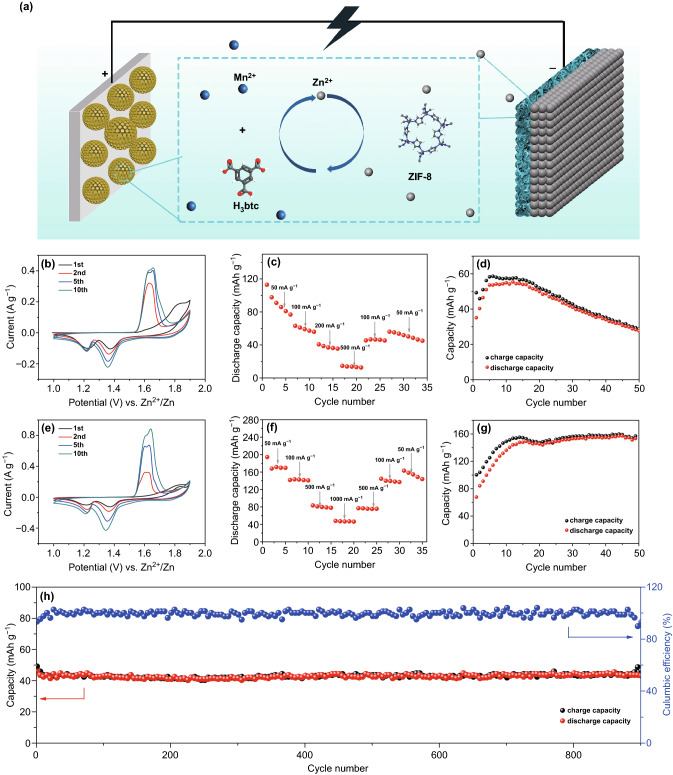
**a** Schematic of the Mn(BTC) cathode//ZIF-8@Zn anode ZIBs. Electrochemical performance of the ZIBs: **b** CV curves at 0.5 mV s^−1^, **c** rate capability, and **d** cycling performance at 100 mA g^−1^ in 2 M ZnSO_4_ electrolyte; **e** CV curves at 0.5 mV s^−1^, **f** rate capability and cycling performance at **g** at 100 mA g^−1^, and **h** at 1000 mA g^−1^ in 2 M ZnSO_4_ + 0.1 M MnSO_4_ electrolyte

An aqueous zinc-ion battery using the above-studied Mn(BTC) cathode and ZIF-8@Zn anode was constructed, as illustrated in Fig. 7a. 2 M ZnSO_4_ aqueous solution was chosen as the electrolyte. CV curves at a scan rate of 0.5 mV s^−1^ are displayed in Fig. 7b. It can be seen that there emerge two reduction peaks at 1.22 and 1.37 V and one oxidation peak at 1.83 V in the initial cycle, and the oxidation peak is corresponding to the oxidation reaction from Mn(BTC) to MnO_2_ as discussed above. From the second cycles, two pairs of reversible redox peaks appear, indicating a reversible Zn^2+^ storage/release process. The rate performance is displayed in Fig. 7c. The battery delivers a discharge capacity of 112, 63, 40, and 14 mAh g^−1^, respectively, at current densities of 50, 100, 200, and 500 mA g^−1^. Figure 7d shows cycling performance of the battery. It can be observed that the Mn(BTC) exhibits a discharge capacity of about 55 mAh g^−1^ at a current density of 100 mA g^−1^ after activation process. However, the capacity rapidly fades to only 27 mAh g^−1^ after 50 charge/discharge cycles, indicating a poor cycling stability. As investigated above, such a poor cycling performance can be mainly ascribed to the dissolution of MnO_2_ from Mn(BTC) cathode. Therefore, to optimize cycling performance of the Mn(BTC) cathode and the assembled battery, MnSO_4_ was added to the ZnSO_4_ electrolyte, because Mn^2+^ in the electrolyte is able to suppress manganese dissolution [78, 79]. In 2 M ZnSO_4_ + 0.1 M MnSO_4_ electrolyte, their electrochemical performance is significantly improved. As shown in Fig. 7e, peak currents of CV curves with the addition of Mn^2+^ in the electrolyte are much higher than that without Mn^2+^ (Fig. 7b), which indicates a better Zn^2+^-storage ability. A superior rate capability is also achieved in Fig. 7f, with the capacity of 170, 142, 80, and 46 mAh g^−1^ at current densities of 50, 100, 500, and 1000 mA g^−1^, respectively. The high rate performance could be ascribed to the stabilization and excellent kinetics of the cathode under the case of Mn^2+^ addition in ZnSO_4_ electrolyte [15]. Cycling performance test (Fig. 7g) shows that the battery delivers a reversible capacity of 150 mAh g^−1^ after 50 cycles at 0.1 A g^−1^. Furthermore, the battery exhibits 92% capacity retention after 900 cycles at 1000 mA g^−1^ in Fig. 7h, which indicates excellent long-cycling stability. It is worth noting that the cycle performance of Mn(BTC) cathode in ZnSO_4_ + MnSO_4_ electrolyte is superior to many previously reported manganese oxide cathodes shown in Table S2 at similar current densities, but the capacity and rate capability are not as good as widely studied MnO_2_ materials. Despite this, MOF cathode materials are still promising for Zn^2+^ storage because further research may find some Mn-MOF or other MOF materials with much better Zn^2+^-storage ability, considering that MOF materials possess diverse structure, controllable chemical composition, very high specific surface area, and many other merits. CV curves (Fig. S11a) at various scan rates were also recorded and used to analyze Zn^2+^ storage kinetics of the cathode. When the scan rate increases from 0.5 to 5 mV s^−1^, both anodic and cathodic peaks slightly shift, which is due to increased polarization at higher scan rates [80]. According to the previous literature, the relationship between peak current (*i*) and the scan rate (*v*) can be represented by Eqs. 1 and 2 [81]:

1 $$i = av^{b}$$

2 $$\log \,i = b\,\log \,v + \log \,a$$

where *a* and *b* are adjustable parameters. In general, *b* value ranges from 0.5 to 1.0. The coefficient *b* of 0.5 indicates a faradic reaction, and the coefficient *b* of 1.0 means a complete surface-controlled capacitive process [82]. The *b* values calculated by slope of the log(*v*)-log(*i*) plots (Fig. S11b) for redox peaks (Fig. S11a) are 0.54 and 0.67. This indicates that Zn^2+^ storage in the Mn(BTC) cathode is mainly through faradic reactions.

## Conclusions

In summary, a high-performance aqueous zinc-ion battery system was realized based on MOF materials. Several kinds of MOF materials were synthesized first and investigated as cathode materials for ZIBs. During them, Mn(BTC) MOF showed a high capacity and its Zn^2+^ storage mechanism was revealed such as a transformation reaction from Mn(BTC) to MnO_2_ during charge process. In addition, a porous ZIF-8 coating was utilized to protect Zn foil anodes, which led to a uniform electrolyte flux and significantly inhibited the formation of zinc protuberances/dendrites. An aqueous zinc-ion battery was constructed based on the Mn(BTC) cathode and the ZIF-8 stabilized Zn anode. Benefiting from the synergetic effect of Mn(BTC) cathode and Mn^2+^ additive in the electrolyte, high capacity and excellent long-term cycling ability were simultaneously realized. This work proves that by exploring MOF materials, high-performance aqueous zinc-ion batteries can be achieved.

## Electronic supplementary material

Below is the link to the electronic supplementary material.

Supplementary material 1 (PDF 1396 kb)
