# Supplementary material for: High-Performance Aqueous Zinc-Ion Batteries Realized by MOF Materials
Source: Nanomicro Lett. 2020 Jul 17;12:152. doi: 10.1007/s40820-020-00487-1 (PMC7770854; doi:10.1007/s40820-020-00487-1)
Supplement: Supplementary file 1 — Supplementary material 1 (PDF 1396 kb) [file 40820_2020_487_MOESM1_ESM.pdf]

Supporting Information for

High-Performance Aqueous Zinc-Ion Batteries Realized by MOF Materials

Xuechao Pu[1, 2], Baozheng Jiang[1, 3], Xianli Wang[1], Wenbao Liu[1, 2], Liubing Dong[4, *], Feiyu Kang[1, 2], Chengjun Xu[1, *]

[1]Shenzhen Geim Graphene Center, Tsinghua Shenzhen International Graduate School, Tsinghua University, Shenzhen 518055, People's Republic of China

[2]State Key Laboratory of New Ceramics and Fine Processing, School of Materials Science and Engineering, Tsinghua University, Beijing 100084, People's Republic of China

[3]Tsinghua-Berkeley Shenzhen Institute (TBSI), Tsinghua University, Shenzhen 518055, People's Republic of China

[4]College of Chemistry and Materials Science, Jinan University, Guangzhou 511443, People's Republic of China

*Corresponding authors. E-mail: donglb@jnu.edu.cn (L. Dong); vivaxuchengjun@163.com (C. Xu)

Supplementary Figures and Tables

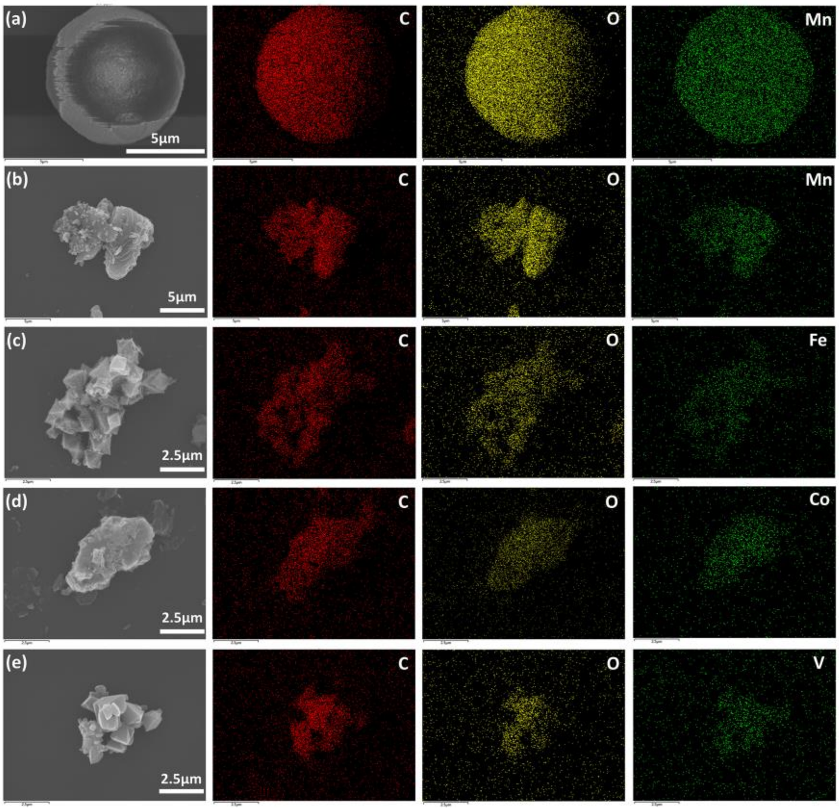

Fig. S1 EDS images of **(a)** Mn(BTC), **(b)** Mn(BDC), **(c)** Fe(BDC), **(d)** Co(BDC), and **(e)** V(BDC) particles

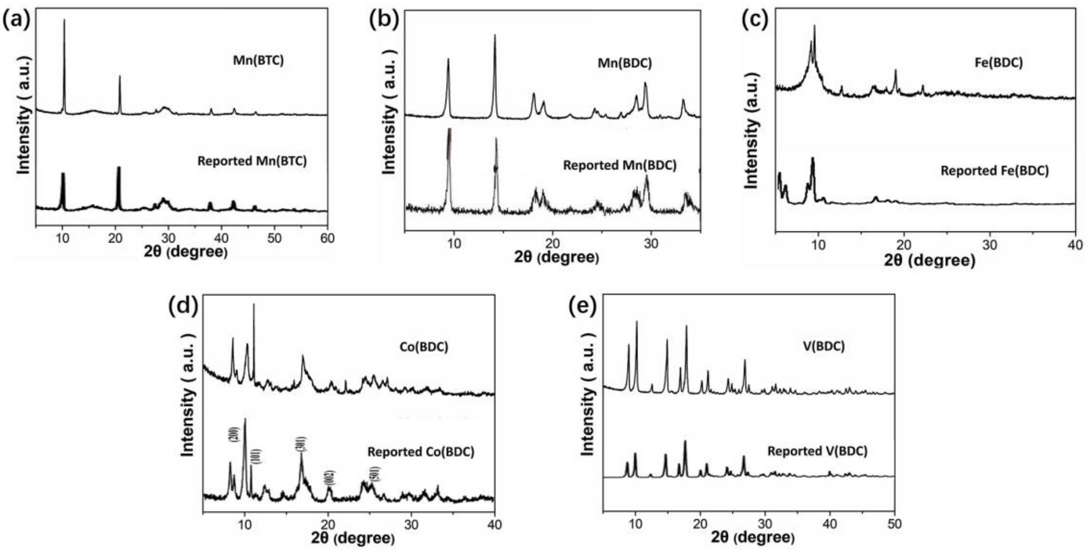

Fig. S2 XRD patterns comparison of as-prepared MOFs and the literature reported MOFs: **(a)** Mn(BTC), **(b)** Mn(BDC), **(c)** Fe(BDC), **(d)** Co(BDC), and **(e)** V(BDC) [S1-S5]

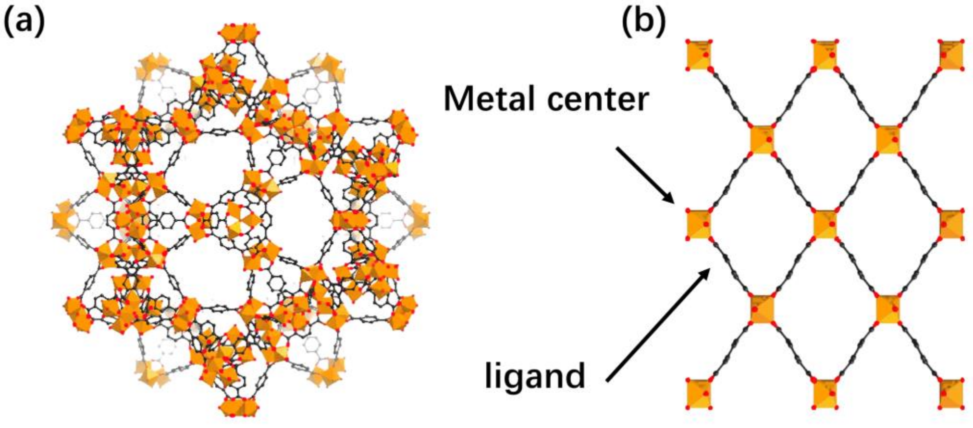

Fig. S3 Crystal structures of synthesized MOFs materials: **(a)** Fe(BDC) and **(b)** V(BDC). The crystalline structures of Mn(BTC), Mn(BDC) and Co(BDC) remain unknown

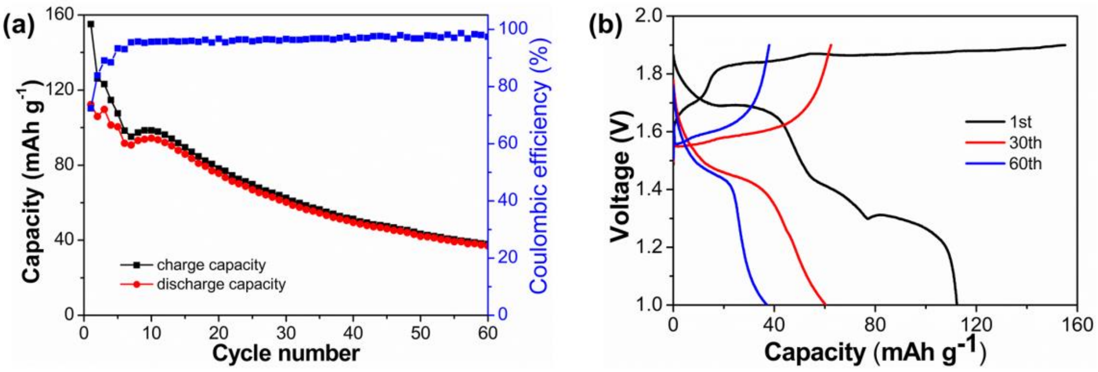

Fig. S4 Electrochemical performance of the Mn(BTC) cathode in 2 M $ZnSO_4$ aqueous electrolyte: **(a)** cycling performance and **(b)** charge/discharge profiles at various cycles at 50 mA g^{-1}

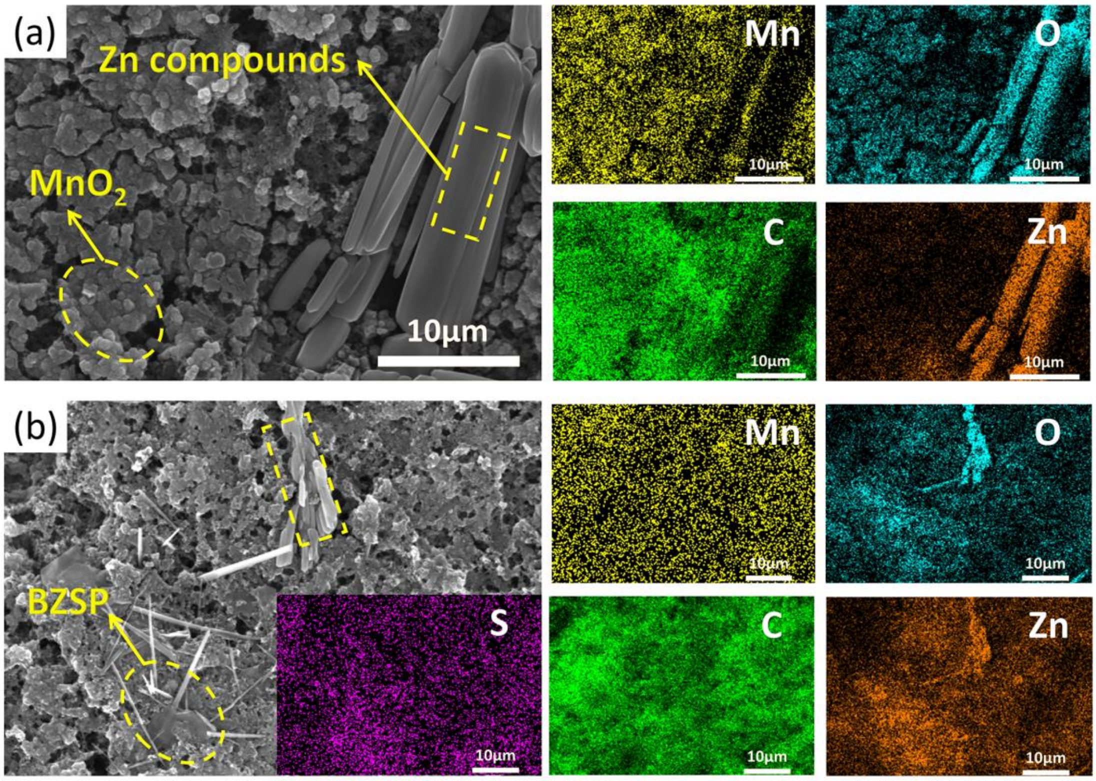

Fig. S5 SEM images and EDS mapping of Mn(BTC) cathode at different charge/discharge states: (a) charge to 1.9 V and (b) then discharge to 1.0 V.

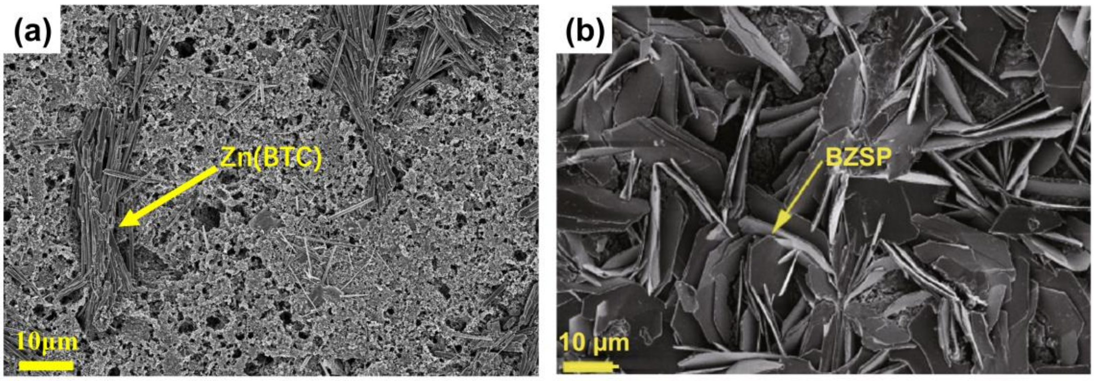

Fig. S6 SEM images of **(a)** Mn(BTC) cathode and **(b)** previously reported MnO_2 cathode at fully discharged states [S6]

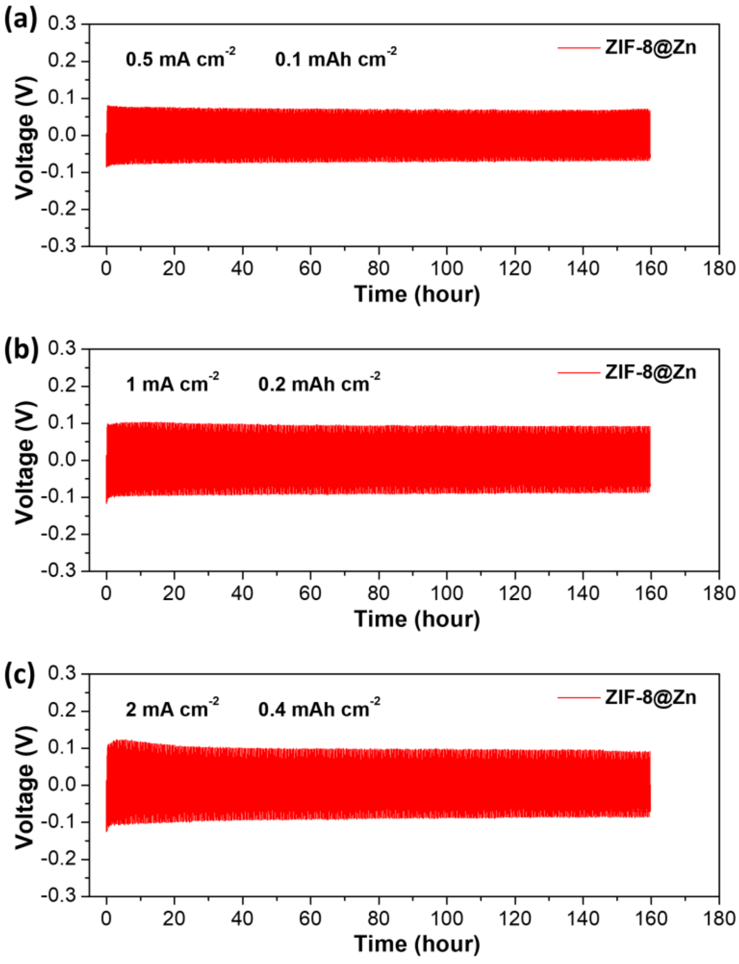

Fig. S7 Cycling performance of ZIF-8@Zn||ZIF-8@Zn symmetrical batteries at different current densities of 0.5~2 mA cm^{-2} and different charge/discharge capacities of 0.1~0.4 mAh cm^{-2} in 2 M $ZnSO_4$ electrolyte

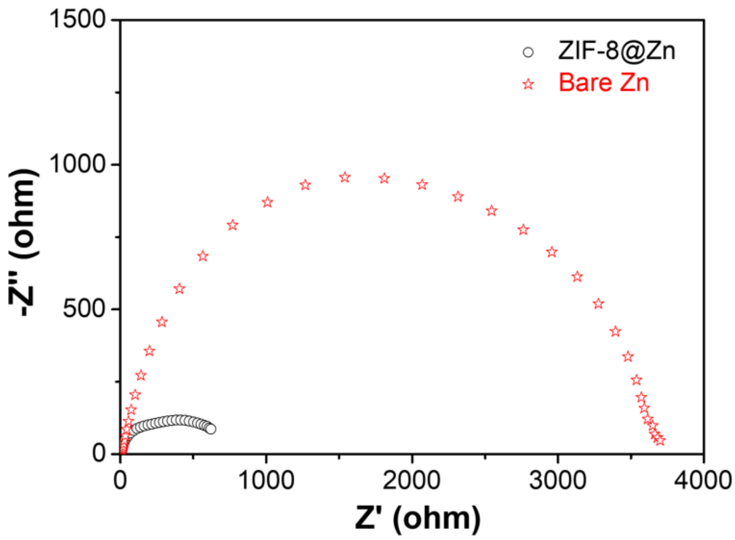

Fig. S8 Nyquist plots of bare Zn and ZIF-8@Zn electrodes based symmetric cells

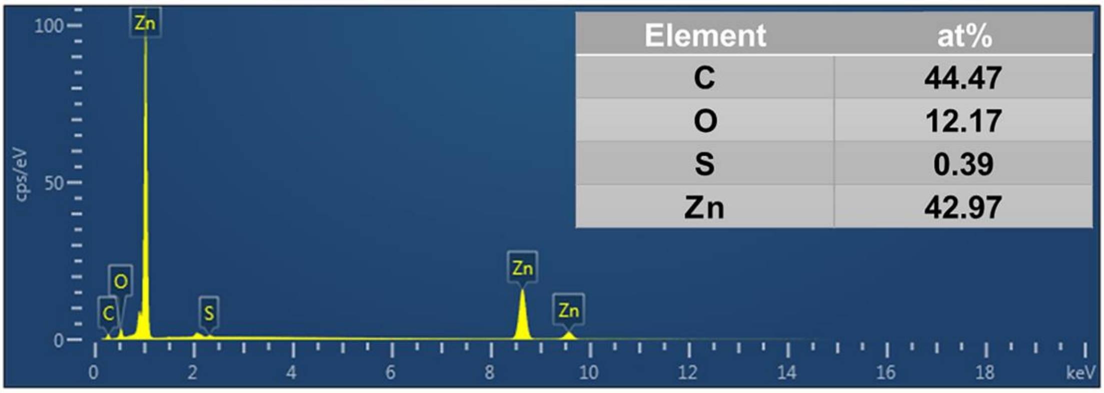

Fig. S9 SEM-EDS result of bare Zn foil electrode after 100 stripping/plating cycles

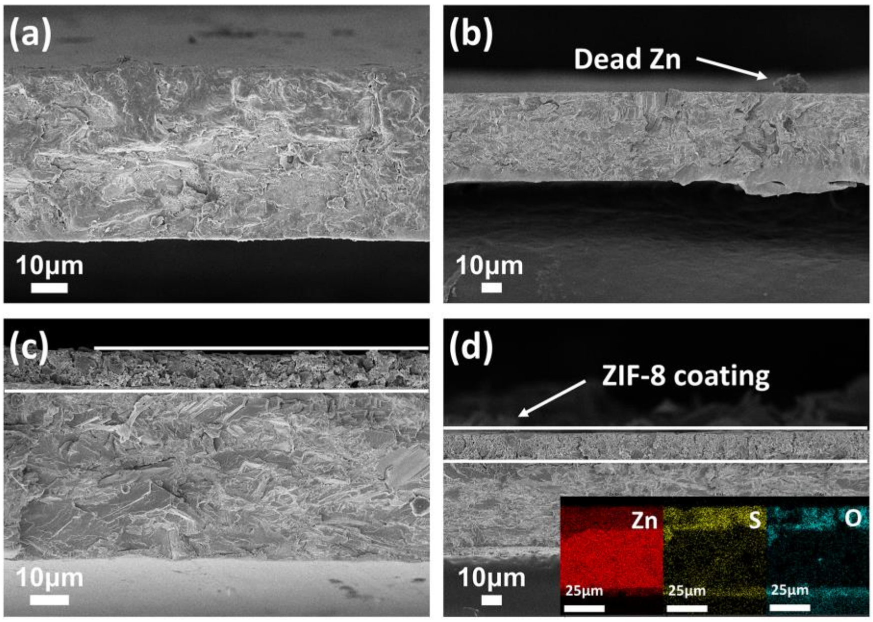

Fig. S10 Cross-sectional SEM images of bare Zn electrode **(a)** before and **(b)** after 100 stripping/plating cycles. Cross-sectional SEM images of ZIF-8@Zn electrode **(c)** before and **(d)** after 100 stripping/plating cycles. Inset in **(d)** is EDS mapping of the cross-section

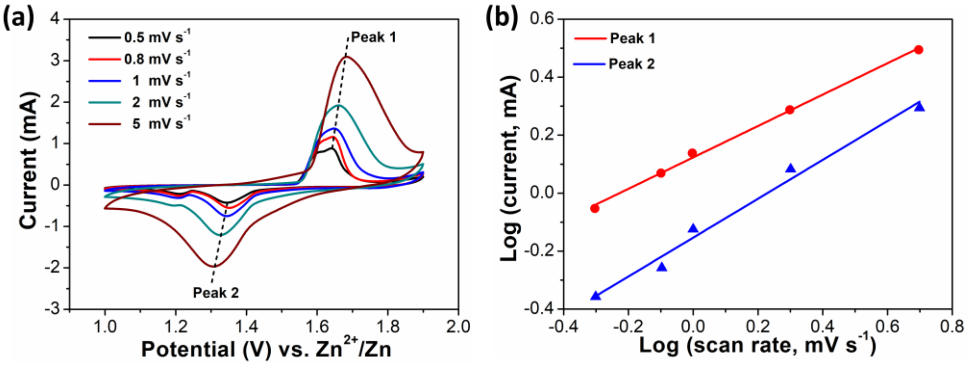

Fig. S11 (a) CV curves of Mn(BTC) cathode//ZIF-8@Zn anode ZIBs in 2 M $ZnSO_4$ + 0.1 M $MnSO_4$ electrolyte at scan rates of 0.5, 0.8, 1, 2 and 5 mV s^{-1}; **(b)** Dependence of log(v) and log(i) at various scan rates ranging from 0.5 to 5 mV s^{-1}

Table S1 Plasma atomic emission spectrometry (ICP-AES) results

Electrolyte of different samples	Mn content (mg L^{-1})
Original cathode	≤0.05
Cathode charged to 1.8 V	6.15
Cathode charged to 1.9 V	4.62

Table S2 Comparison of cycling performance of different cathode materials for ZIBs

Samples	Electrolyte	Cycling performance	Test current	Ref.
α-MnO_2	1 M $ZnSO_4$	63% retained after 50 cycles	83 mA g^{-1}	[S7]
α-MnO_2	1 M $ZnSO_4$	66% retained after 30 cycles	10.5 mA g^{-1}	[S8]
α-MnO_2	1 M $ZnSO_4$	70% retained after 30 cycles	42 mA g^{-1}	[S9]
δ-MnO_2	1 M $ZnSO_4$	46% retained after 100 cycles	83 mA g^{-1}	[S10]
α-MnO_2 @CNT	2 M $ZnSO_4$ + 0.5 M $MnSO_4$	99% retained after 500 cycles	5000 mA g^{-1}	[S11]
MnO_2	0.25 M $ZnSO_4$ + 0.75 M Na_2SO_4	53% retained after 1000 cycles	3080 mA g^{-1}	[S12]
α-Mn_2O_3	2 M $ZnSO_4$ + 0.1 M $MnSO_4$	51% retained after 2000 cycles	2000 mA g^{-1}	[S13]
Spinel Mn_3O_4	2 M $ZnSO_4$	73% retained after 300 cycles	500 mA g^{-1}	[S14]
$ZnMn_2O_4$ @C	3 M $Zn(CF_3SO_3)_2$	94% retained after 500 cycles	500 mA g^{-1}	[S15]
β-MnO_2	1 M $ZnSO_4$ + 0.1 M $MnSO_4$	75% retained after 200 cycles	200 mA g^{-1}	[S16]
Mn(BTC)	2 M $ZnSO_4$ + 0.1 M $MnSO_4$	92% retained after 900 cycles	1000 mA g^{-1}	This work

Supplementary References

[S1] Z. Cui, Q. Liu, C. Xu, R. Zou, J. Zhang et al., A new strategy to effectively alleviate volume expansion and enhance the conductivity of hierarchical MnO@C nanocomposites for lithium ion batteries. J. Mater. Chem. A. **5**(41), 21699-21708 (2017). https://doi.org/10.1039/c7ta05986h

[S2] H. Hu, X. Lou, C. Li, X. Hu, T. Li et al., A thermally activated manganese 1,4-benzenedicarboxylate metal organic framework with high anodic capability for Li-ion batteries. New J. Chem. **40**(11), 9746-9752 (2016). https://doi.org/10.1039/c6nj02179d

[S3] K. M. L. Taylor-Pashow, J. D. Rocca, Z. Xie, S. Tran, W. Lin, Postsynthetic

modifications of iron-carboxylate nanoscale metal-organic frameworks for imaging and drug delivery. J. Am. Chem. Soc. **131**(40), 14261-14263 (2009). https://doi.org/10.1021/ja906198y

[S4] X. Hu, H. Hu, C. Li, T. Li, X. Lou et al., Cobalt-based metal organic framework with superior lithium anodic performance. J. Solid State Chem. **242**, 71-76 (2016). https://doi.org/10.1016/j.jssc.2016.07.021

[S5] T. Kim, H. Kim, T.-S. You, J. Kim, Carbon-coated V_2O_5 nanoparticles derived from metal-organic frameworks as a cathode material for rechargeable lithium-ion batteries. J. Alloys Compd. **727**, 522-530 (2017). https://doi.org/10.1016/j.jallcom.2017.08.179

[S6] Y. Huang, J. Mou, W. Liu, X. Wang, L. Dong et al., Novel insights into energy storage mechanism of aqueous rechargeable Zn/MnO_2 batteries with participation of Mn^{2+}. Nano-Micro Lett. **11**(1), 49 (2019). https://doi.org/10.1007/s40820-019-0278-9

[S7] M. H. Alfaruqi, J. Gim, S. Kim, J. Song, J. Jo et al., Enhanced reversible divalent zinc storage in a structurally stable α-MnO_2 nanorod electrode. J. Power Sources. **288**, 320-327 (2015). https://doi.org/10.1016/j.jpowsour.2015.04.140

[S8] B. Lee, C. S. Yoon, H. R. Lee, K. Y. Chung, B. W. Cho et al., Electrochemically-induced reversible transition from the tunneled to layered polymorphs of manganese dioxide. Sci. Rep. **4**, 6066 (2014). https://doi.org/10.1038/srep06066

[S9] B. Lee, H. R. Lee, H. Kim, K. Y. Chung, B. W. Cho et al., Elucidating the intercalation mechanism of zinc ions into α-MnO_2 for rechargeable zinc batteries. Chem. Commun. **51**, 9265-9268 (2015). https://doi.org/10.1039/C5CC02585K

[S10] M. H. Alfaruqi, J. Gim, S. Kim, J. Song, D. T. Pham et al., A layered δ-MnO_2 nanoflake cathode with high zinc-storage capacities for eco-friendly battery applications. Electrochem. Commun. **60**, 121-125 (2015). https://doi.org/10.1016/j.elecom.2015.08.019

[S11] D. Xu, B. Li, C. Wei, Y.-B. He, H. Du et al., Preparation and characterization of MnO_2/acid-treated CNT nanocomposites for energy storage with zinc ions. Electrochim. Acta. **133**, 254-261 (2014). https://doi.org/10.1016/j.electacta.2014.04.001

[S12] J. S. Ko, M. B. Sassin, J. F. Parker, D. R. Rolison, Jeffrey W. Long, Combining battery-like and pseudocapacitive charge storage in 3D MnO_x@carbon electrode architectures for zinc-ion cells. Sustain. Energy Fuels. **2**(3), 626-636 (2018). https://doi.org/10.1039/c7se00540g

[S13] B. Jiang, C. Xu, C. Wu, L. Dong, J. Li et al., Manganese sesquioxide as cathode material for multivalent zinc ion battery with high capacity and long cycle life. Electrochim. Acta. **229**, 422-428 (2017). https://doi.org/10.1016/j.electacta.2017.01.163

[S14] J. Hao, J. Mou, J. Zhang, L. Dong, W. Liu et al., Electrochemically induced spinel-layered phase transition of Mn_3O_4 in high performance neutral aqueous rechargeable zinc battery. Electrochim. Acta. **259**, 170-178 (2018). https://doi.org/10.1016/j.electacta.2017.10.166

[S15] N. Zhang, F. Cheng, Y. Liu, Q. Zhao, K. Lei et al., Cation-deficient spinel $ZnMn_2O_4$ cathode in $Zn(CF_3SO_3)_2$ electrolyte for rechargeable aqueous Zn-ion battery. J Am. Chem.

Soc. **138**(39), 12894-12901 (2016). https://doi.org/10.1021/jacs.6b05958

[S16] S. Islam, M. H. Alfaruqi, V. Mathew, J. Song, S. Kim et al., Facile synthesis and the exploration of the zinc storage mechanism of β-MnO_2 nanorods with exposed (101) planes as a novel cathode material for high performance eco-friendly zinc-ion batteries. J. Mater. Chem. A. **5**(44), 23299-23309 (2017). https://doi.org/10.1039/c7ta07170a
